# PET/CT联合CT三维重建在鉴别早期肺腺癌不同病理亚型中的价值

**DOI:** 10.3779/j.issn.1009-3419.2021.101.19

**Published:** 2021-07-20

**Authors:** 杰 游, 国中 张, 祥龙 高, 勇 陈, 余声 束

**Affiliations:** 225001 扬州，江苏省苏北人民医院胸外科 Department of Thoracic Surgery, Northern Jiangsu People's Hospital, Yangzhou 225001, China

**Keywords:** 部分实性结节, 肺腺癌, CT三维重建, PET/CT, 病理亚型, Part-solid nodules, Lung neoplasms, CT three-dimensional reconstruction, PET/CT, Pathological subtypes

## Abstract

**背景与目的:**

早期肺腺癌中病理亚型以贴壁为主型的浸润性腺癌（lepidic predominant invasive adenocarcinoma, LPA）与原位腺癌（adenocarcinoma *in situ*, AIS）、微浸润性腺癌（microinvasive adenocarcinoma, MIA）的良好预后相似，临床上也迫切需要能够区分LPA与非LPA型浸润性腺癌（non-lepidic predominant invasive adenocarcinoma, non-LPA）的手段，本研究拟通过正电子发射型计算机断层显像（positron emission computed tomography, PET）/计算机断层扫描（computed tomography, CT）的最大标准化摄取值（maximal standard uptake value, SUVmax）和CT三维重建后参数探讨术前影像学表现为部分实性结节（part-solid nodules, PSNs）的早期肺腺癌不同病理亚型间的关系。

**方法:**

回顾性分析2016年1月-2019年1月于江苏省苏北人民医院胸外科行解剖性肺切除术且影像学表现为PSNs的早期肺腺癌患者资料，所有患者胸部增强CT和PET/CT资料完整可获取，利用Mimics软件行三维重建，获取肿瘤体积、肿瘤三维平均CT值（3-dimensional mean-CT value, 3Dm-CT）、SUVmax等数据，采用SPSS 25.0进行统计分析，GraphPad Prism 8.3.0绘制受试者工作曲线（receiver operating curve, ROC），*P* < 0.05为差异有统计学意义。

**结果:**

最终共计67例患者纳入本研究，按病理亚型不同将所有患者分为两组，AIS、MIA及浸润性腺癌（invasive adenocarcinoma, IAC）中的LPA归为低危组28例（41.8%），其余non-LPA如腺泡型（acinar pattern-predominant adeno-carcinoma, APA）、乳头型（papillary pattern-predominant adenocarcinoma, PPA）、微乳头型（micropapillary pattern-predominant adeno-carcinoma, MPA）归为高危组39例（58.2%），两组间SUVmax（*t*=3.153, *P*=0.002）、肿瘤体积（*t*=3.331, *P*=0.001)、实性/磨玻璃成分体积（*t*=2.74, *P*=0.006) /（*t*=3.127, *P*=0.002）、实性/磨玻璃成分3Dm-CT（*t*=3.655, *P* < 0.001) /（*t*=7.082, *P* < 0.001) 均具有显著统计学意义。ROC曲线提示：SUVmax[曲线下面积（area under curve, AUC）=0.727]、肿瘤体积（AUC=0.740）、磨玻璃成分体积（AUC=0.725）、实性成分3Dm-CT（AUC=0.763）、磨玻璃成分3Dm-CT（AUC=0.756）预测效能最佳。将上述AUC > 0.7的协变量纳入多因素ROC曲线分析，获得联合预测因子（AUC=0.835）具有中等以上预测价值。

**结论:**

PET/CT中SUVmax和CT三维重建参数与影像学表现为PSNs的早期肺腺癌的不同病理亚型具有显著相关性，联合SUVmax、肿瘤体积、磨玻璃成分体积和实性/磨玻璃成分3Dm-CT对鉴别表现为PSNs的早期肺腺癌的病理亚型具有一定价值。

## Abstract (en)

**Background and objective:**

The good prognosis of lepidic predominant invasive adenocarcinoma (LPA) and adenocarcinoma *in situ* (AIS)/microinvasive adenocarcinoma (MIA) in the pathological subtypes of early lung adenocarcinoma is similar, and the means to distinguish LPA from non-LPA is urgently needed in clinical practice. This study intends to analyze the correlation between positron emission computed tomography (PET)/computed tomography (CT) maximal standard uptake value (SUVmax) with CT three-dimensional reconstruction parameters and the pathological subtypes of early lung adenocarcinoma with part-solid nodules (PSNs) in preoperative imaging.

**Methods:**

The data of early lung adenocarcinoma patients who underwent anatomical pneumonectomy at the Department of Thoracic Surgery of Northern Jiangsu People's Hospital from January 2016 to January 2019 retrospectively analyzed and subsolid nodules on imaging were showed. All patients with enhanced chest CT and PET/CT data can be obtained completely, using Mimics software to perform three-dimensional reconstruction to obtain tumor volume, 3-dimensional mean-CT value (3Dm-CT) of tumor and SUVmax, using SPSS 25.0 for statistical analysis and GraphPad Prism 8.3.0 for drawing receiver operating curve (ROC). *P* < 0.05 indicates that the difference is statistically significant.

**Results:**

67 patients were included in this study. All patients were divided into two groups according to different pathological subtypes. AIS, MIA and LPA in invasive adenocarcinoma (IAC) were in the low-risk group, 28 cases (41.8%), and the remaining non-LPA were in high-risk group, 39 cases (58.2%). SUVmax (*t*=3.153, *P*=0.002), tumor volume (*t*=3.331, *P*=0.001), solid/ground glass component volume (*t*=2.74, *P*=0.006)/(*t*=3.127, *P*=0.002) and 3Dm-CT of solid/ground glass component (*t*=3.655, *P* < 0.001)/(*t*=7.082, *P* < 0.001) between the two groups were all statistically significant. ROC curve prompts: SUVmax [area under curve (AUC)=0.727], tumor volume (AUC=0.740), ground glass component volume (AUC=0.725), 3Dm-CT of solid components (AUC=0.763), 3Dm-CT of ground glass components (AUC=0.756) have the best predictive performance. The above-mentioned covariates with AUC > 0.7 were included in the multivariate ROC curve analysis, and the joint predictor (AUC=0.835) was obtained with medium or above predictive value.

**Conclusion:**

PET/CT SUVmax and CT three-dimensional reconstruction parameters have a significant correlation with the different pathological subtypes of early lung adenocarcinoma with PSNs in imaging. The combination of SUVmax, tumor volume, ground glass component volume and 3Dm-CT of solid/ground glass component CT value has certain value in identifying the pathological subtype of early stage lung adenocarcinoma with PSNs nodules in imaging.

2020年我国肺癌新发病数、死亡例数分别占所有癌症的21.8%、23.8%，位居所有癌症发病的首位，并显著高于其他恶性肿瘤^[1]^，随着胸部低剂量计算机断层扫描（low-dose computed tomography, LDCT）的推广普及，越来越多的肺部早期病变得以发现，尤其自去年新型冠状病毒疫情爆发以来，胸部LDCT筛查成为常态，使得肺结节的检出率迅猛增长。而在这些检出的肺结节中，存在许多部分实性结节（part-solid nodules, PSNs），即介于纯实性与纯磨玻璃样结节之间，实性与磨玻璃两种成分同时存在的一种影像学表现。PSNs的术后病理往往被证实为肺腺癌，根据2011年国际肺癌研究协会（International Association for the Study of Lung Cancer, IASLC）/美国胸科学会（American Thoracic Society, ATS）/欧洲呼吸学会（European Respiratory Society, ERS），以及2015年世界卫生组织（World Health Organization, WHO）的分类定义，将腺癌分为原位腺癌（adenocarcinoma *in situ*, AIS）、微浸润腺癌（microinvasive adenocarcinoma, MIA）、浸润性腺癌（lepidic predominant invasive adenocarcinoma, LPA），其中浸润性腺癌（invasive adenocarcinoma, IAC）又分贴壁型（lepidic predominant invasive adenocarcinoma, LPA）、腺泡型（acinar pattern-predominant adenocarcinoma, APA）、乳头型（papillary pattern-predominant adenocarcinoma, PPA）、微乳头型（micropapillary pattern-predominant adenocarcinoma, MPA）、实体型（solid pattern-predominant adenocarcinoma, SPA）等病理亚型^[2, 3]^。而病理亚型的不同往往与早期肺腺癌患者的预后显著相关^[4, 5]^。Murakami等^[6]^根据上述肺腺癌分类方法，回顾了347例cIa期肺腺癌患者，发现AIS、MIA、LPA、APA、PPA、SPA和MPA七种病理亚型的5年无病生存率分别为100%、100%、99%、82.4%、80.8%、73.6%和33.3%。Russell等^[7]^随访了210例早期肺腺癌患者，也得到相似结果。影像学中，上述病理亚型均可以表现为PSNs，但以LPA为界，LPA与非LPA型浸润性腺癌（non-lepidic predominant invasive adenocarcinoma, non-LPA）的预后差异巨大，因此临床上迫切需要术前能够预测两者的手段，以制定更恰当的围术期诊疗策略。

本研究拟通过正电子发射型计算机断层显像（positron emission computed tomography, PET）/CT的最大标准化摄取值（maximal standard uptake value, SUVmax）和CT三维重建后PSNs所表现出的特征参数，探讨其在影像学表现为PSNs早期肺腺癌不同病理亚型中的应用价值。

## 1 资料与方法

### 1.1 一般资料

选取2016年1月-2019年1月就诊于江苏省苏北人民医院胸外科、术前胸部增强CT表现为PSNs并同期行PET/CT检查的患者191例。纳入标准：术后病理证实为肺腺癌、临床分期为Ia期者。排除标准：既往有肺结核病史、恶性肿瘤病史、远处转移、近期肺炎病史及其他资料不全者。最终符合标准的患者共计67例，同时收集患者的临床病例资料，包括性别、年龄、基础病史、吸烟史、术后病理、术前增强CT、PET/CT影像编号等。将所有患者按病理亚型不同分为两组，鉴于LPA虽属IAC但其术后5年总生存率、无病生存率与AIS、MIA相似均接近100%，因此将AIS、MIA、LPA归为低危组，APA、PPA、MPA归为高危组。

### 1.2 检查方法

#### 1.2.1 增强CT

采用美国GE Light Speed 64排螺旋CT机进行增强扫描，扫描参数：球管电压120 kV，管电流150 mA，层厚/间距5.00 mm，螺距1.375。辅助患者取仰卧位，双手抱头以暴露胸部，嘱吸气后屏气。由肺尖至肋膈角常规CT轴面平扫和增强扫描。平扫完成后予高压注射器于患者外周静脉注射造影剂碘海醇80 mL-100 mL，流率3 mL/s，再对肺内结节实施动态增强扫描。肺窗：窗位-700 Hu，窗宽1, 000 Hu，纵隔窗：窗位40 Hu，窗宽350 Hu。扫描后重建成1.0 mm-1.25 mm的薄层图像，传至PACS系统进行图像分析。

#### 1.2.2 PET/CT

采用美国GE discovery VCT64型PET/CT进行检查。预约检查前应告知患者检查当天禁食12 h、禁饮6 h，停用含糖药物，且空腹血糖应 < 10 mmol/L。肘部静脉注射^18^F-FDG示踪剂，注射剂量按3.14 MBq/kg-5.11 MBq/kg计算，给药后休息60 min排尿后开始全身CT及PET扫描。全上下半身成像覆盖区域从颅顶至臀部，胸部扫描的范围从锁骨上窝区至双肾上级水平，采集6个-8个床位。PET/CT图像的判读采用视觉分析法和半定量分析法相结合，机器自动选定异常区域辅以人工手动修正，测量感兴趣区域内最大SUV值，即为SUVmax。

### 1.3 三维重建方法

采用Mimics Medical 21.0软件进行肺结节三维重建处理。本研究经江苏省苏北人民医院科技处批准，通过收集的术前增强CT影像编号从PACS系统下载入组患者的增强CT薄层图像（DICOM格式）用于三维重建成像。于软件内取肺窗：窗位-700 Hu，窗宽1, 000 Hu，定位结节所在层面，框定出结节大致边界，影像医师逐层修改并确定界内区域，采用软件Dynamic Region Grow功能，误差值设定为（-100 Hu, 100 Hu），行区域内血管自动扩增重建，取该患者重建后区域内血管CT值所在区间的最小值作为PSNs实性成分上限值（以屏蔽血管对体积测量的干扰），采用相同方法对区域内实性成分及磨玻璃成分分别行自动扩增重建，选取实性成分CT值区间内最小值为实性成分下限，同时为磨玻璃成分上限；选取磨玻璃成分最小值为磨玻璃成分下限。以上述3类最小值作为区分实性、磨玻璃成分的上下限阈值，再运用软件中New Mask功能分别重建实性、磨玻璃成分。最后通过读取Mask Properties可直接获得两种成分各自体积与三维平均CT值（3-dimensional mean-CT value, 3Dm-CT），将两种成分体积相加则获得肿瘤体积，最终数据由两位胸外科医师分别行三维重建取两次数值的平均值，重建结果样图如图 1。

**图 1:**
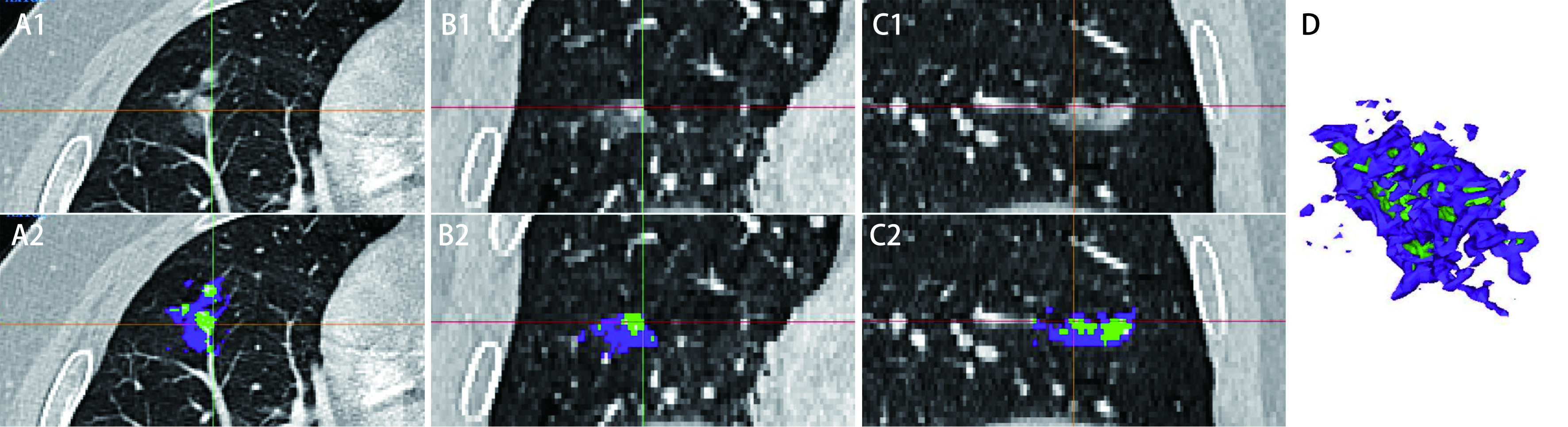
CT三维重建示例。A：轴面；B：冠状面；C：矢状面；D：三维成像。病例来源：63岁老年男性患者，病灶位于右肺中叶，影像学表现为PSNs。重建后参数：肿瘤体积=1, 294 mm^3^，实性/磨玻璃成分体积=269 mm^3^/1, 025 mm^3^，实性/磨玻璃成分3Dm-CT=-5 Hu/-388 Hu，SUVmax=2.97，术后病理亚型为PPA。 CT 3D reconstruction example. A: axial plane; B: coronal plane; C: sagittal plane; D: 3D imaging. Case source: 63-years-old, male patient, PSNs in the right middle lobe. Parameters after reconstruction: tumor volume=1, 294 mm^3^, solid/ground glass component volume=269 mm^3^/1, 025 mm^3^, solid/ground glass component 3Dm-CT: -5 Hu/-388 Hu. SUVmax=2.97, the postoperative pathological subtype was PPA. PSNs: part-solid nodules; 3Dm-CT: 3-dimensional mean-CT value; SUVmax: maximal standard uptake value; PPA: papillary pattern-predominant adenocarcinoma.

### 1.4 统计学方法

采用SPSS 25.0进行统计分析。连续变量以均数±标准差（Mean±SD）表示，其中符合正态分布使用独立样本*t*检验；不符合正态分布则使用非参数检验。分类变量以频数（百分数）表示，使用卡方检验和*Fisher*精确检验。采用GraphPad Prism 8.3.0软件绘制受试者工作曲线（receiver operating curve, ROC）并计算ROC曲线下面积（area under curve, AUC），采用二元*Logistic*回归方程整合AUC > 0.7的协变量，将整合后的联合预测因子再次进行ROC曲线分析，得出联合预测效能。本研究中以AUC > 0.7作为中等预测效能，AUC > 0.9作为高预测效能。*P* < 0.05为差异有统计学意义。

## 2 结果

### 2.1 一般资料

患者一般资料如表 1所示，共计67例，男性23例，女性44例，年龄（54.31±12.02）岁。而病理亚型为AIS 2例（3.0%），MIA 18例（26.9%），IAC 47例（70.1%），其中LPA 8例（11.9%），APA 29例（43.3%），PPA 9例（13.4%），MPA 1例（1.5%）。将67例病例按低危和高危分组后得到，低危组28例（41.8%），高危组39例（58.2%）。其中性别（χ^2^=0.102, *P*=0.75）、年龄（*t*=3.021, *P*=0.08）、基础病史（χ^2^=2.436, *P*=0.119）、吸烟史（χ^2^=2.431, *P*=0.123）、pT分期（χ^2^=1.286, *P*=0.526）均无统计学差异。

**表 1:** 67例PSNs早期肺腺癌患者一般资料和重建后参数表 General data of 67 patients with early stage lung adenocarcinoma of PSNs and the parameter table after reconstruction

Item	Total	Low-risk	High-risk	x^2^/*t*	*P*
Patients (*n*)	67	28	39		
Gender				0.102	0.75
Male	23 (34.3%)	9 (32.1%)	14 (35.9%)		
Female	44 (65.7%)	19 (67.9%)	25 (64.1%)		
Age (yr)	54.31±12.02	51.32±13.36	56.46±10.61	3.021	0.084
Basic disease				2.436	0.119
Yes	16 (23.9%)	4 (14.3)	12 (30.8%)		
No	51 (76.1%)	24 (85.7)	27 (69.2%）		
Smoking				2.431	0.123
Yes	7 (10.4%)	1 (3.6%)	6 (15.4%)		
No	60 (89.6%)	27 (96.4%)	33 (84.6%)		
pT stage				1.286	0.526
pT1a	15 (22.4%)	8 (28.6%)	7 (17.9%)		
pT1b	37 (55.2%)	15 (53.6%)	22 (56.4%)		
pT1c	15 (22.4%)	5 (17.9%)	10 (25.6%)		
SUVmax	1.52±1.52	0.83±0.59	2.01±1.79	3.153	0.002
Volume (mm^3^)					
T.vol	2, 127.97±2, 054.32	1, 255.36±1, 232.72	2, 754.46±2, 589.73	3.331	0.001
S.vol	662.09±1, 055.77	287.61±315.26	930.95±1, 299.17	2.740	0.006
G.vol	1, 465.88±1, 337.28	967.75±953.38	1, 823.51±1, 464.96	3.127	0.002
3Dm-CT (Hu)					
S.3Dm-CT	-57.00±114.79	-107.29±109.45	-22.42±106.23	3.655	< 0.001
G.3Dm-CT	-485.00±122.15	-545.79±75.90	-441.36±130.93	7.082	< 0.001
Volume ratio (%)					
S.ratio	25.10±17.04	20.13±11.21	28.67±19.60	1.462	0.144
G.ratio	74.90±17.04	79.86±11.21	71.33±19.60	-1.462	0.144
T.vol: tumor volume; S.vol: solid component volume; G.vol: ground glass component volume; S.3Dm-CT: solid component 3-dimensional mean-CT value; G.3Dm-CT: ground glass component 3-dimensional mean-CT value; S.ratio: solid component ratio; G.ratio: ground glass component ratio.					

### 2.2 SUVmax比较

患者SUVmax全组为1.52±1.52，低危组为0.83±0.59，高危组为2.01±1.79，在两组病理类型中具有显著统计学差异（*t*=3.153, *P*=0.002）。

### 2.3 三维重建参数比较

三维重建参数中，肿瘤体积为（2, 127.97±2, 054.32）mm^3^，低危组为（1, 255.36±1, 232.72）mm^3^，高危组为（2, 754.46±2, 589.73）mm^3^。全组实性成分的3Dm-CT为（-57±114.79）Hu，低危组为（-107.29±109.45）Hu，高危组为（-22.42±106.23）Hu；全组磨玻璃成分3Dm-CT为（-485.0±122.1）Hu，低危组为（-545.8±75.9）Hu，高危组为（-441.4±130.9）Hu，通过独立样本*t*检验和非参数检验对比两组数据后，发现肿瘤体积（*t*=3.331, *P*=0.001）、实性成分体积（*t*=2.74, *P*=0.006）、磨玻璃成分体积（*t*=3.127, *P*=0.002）、实性成分3Dm-CT（*t*=3.655, *P* < 0.001）和磨玻璃成分3Dm-CT（*t*=7.082, *P* < 0.001）均具有显著统计学差异，而实性/肿瘤体积比（*t*=1.462, *P*=0.144)、磨玻璃/肿瘤体积比（*t*=-1.462, *P*=0.144）无统计学差异。

### 2.4 单、多协变量预测两组病理亚型的效能

在各具有统计学差异的协变量中，SUVmax（AUC为0.727，灵敏度为61.5%，特异度为82.1%，截断值为1.26）、肿瘤体积（AUC为0.740，灵敏度为64.1%，特异度为78.6%，截断值为1, 473 mm^3^）、磨玻璃体积（AUC为0.725，灵敏度为50%，特异度为84.6%，截断值为472 mm^3^）、实性体积（AUC为0.697，灵敏度为64.1%，特异度为71.4%，截断值为265 mm^3^）、实性3Dm-CT（AUC为0.763，灵敏度为66.7%，特异度为85.7%，截断值为-35 Hu）、磨玻璃3Dm-CT（AUC为0.756，灵敏度为79.5%，特异度为78.6%，截断值为-509 Hu）预测效能最佳。将AUC > 0.7的协变量纳入多因素ROC曲线分析，获得联合预测因子（AUC为0.835，灵敏度为76.9%，特异度为85.7%，截断值为0.55）具有中等以上预测价值（图 2）。

**图 2:**
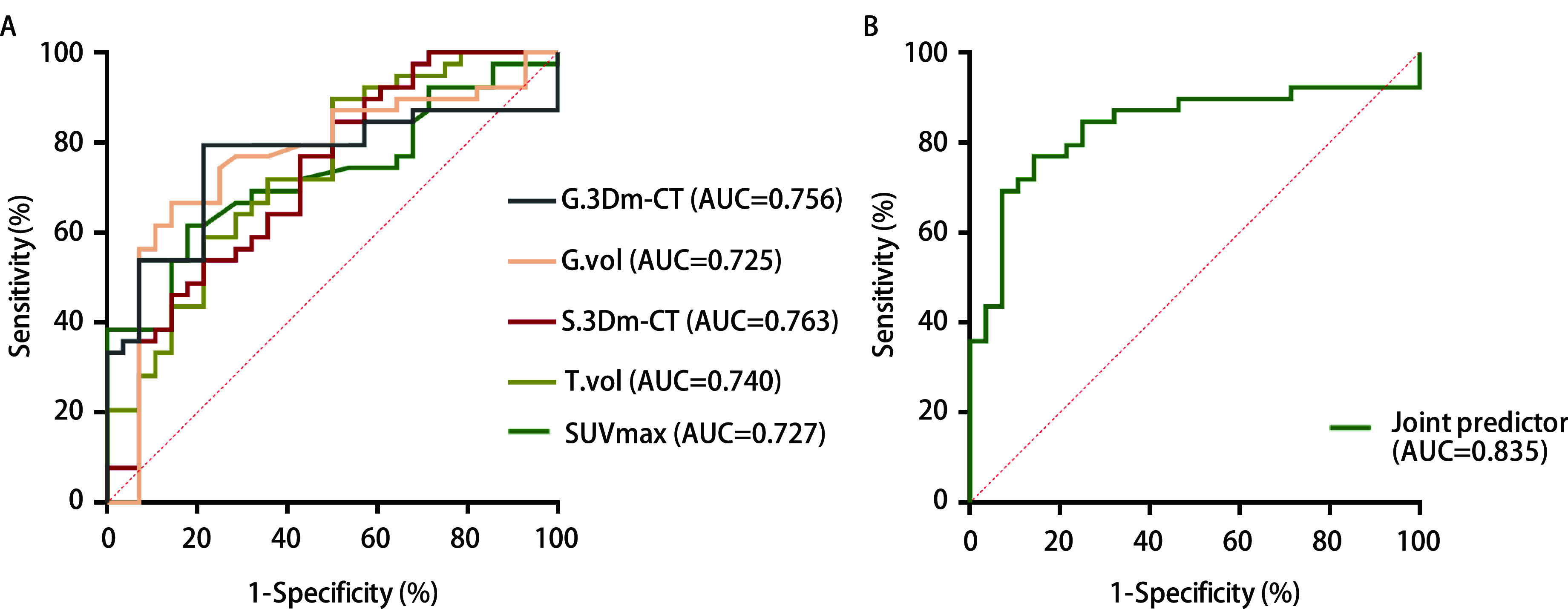
ROC曲线图。A：协变量ROC曲线；B：联合预测ROC曲线。 ROC curve graph. A: covariate ROC curve; B: joint predictor ROC curve. SUVmax: maximal standard uptake value; ROC: receiver operating curve; AUC: area under curve.

## 3 讨论

### 3.1 病理亚型与肿瘤体积的关系

本研究证实影像学表现为PSNs的早期肺腺癌的病理特征与形态特征相关，两组中肿瘤体积、实性和磨玻璃成分体积均存在显著差异，当肿瘤体积 > 1, 473 mm^3^、磨玻璃体积 > 472 mm^3^或实性体积 > 265 mm^3^时需考虑病理亚型为高危组即non-LPA。Kobayashi、Eguchi、Lee等^[8-10]^认为肺腺癌组织学亚型从AIS到IAC的发生发展与肿瘤大小的变化密切相关，瘤体越大，组织病理学亚型进展越晚，两者呈显著线性关系，这与我们的结论一致。在实际临床中，PSNs往往表现为不规则形态，仅利用病灶轴面最大径描述肿瘤大小特征的准确性可能较为局限。本研究利用三维重建软件计算肿瘤体积，整合了肿瘤长宽高三个维度，在肿瘤大小这一特征的描述上，更为接近肿瘤的真实情况。在PSNs中，实性成分和磨玻璃成分的不规则形态较瘤体本身更为普遍，因此采用三维成像参数描述PSNs特征的优势更为明显。

目前常用最大实性/最大肿瘤直径比（consolidation-tumor ratio, CTR）作为早期PSNs肺腺癌实性成分的肿瘤特征。Eriguchi等^[11]^通过联合CTR、SUVmax和CT值可区分LPA和non-LPA。而本研究中，实性/肿瘤体积比（*t*=1.462, *P*=0.144）、磨玻璃/肿瘤体积比（*t*=-1.462, *P*=0.144），在两组中无统计学差异，且磨玻璃体积 > 实性体积的PSNs病例数在两组中均有较高比例。在三维成像过程中，作者发现某些PSNs即使轴面CTR接近1，但三维成像后的体积数据中，磨玻璃成分体积仍可能等于或大于实性成分体积。可能原因考虑为：①实性成分生长不均，仅在少数层面存在；②PSNs大多表现为磨玻璃成分包裹实性成分。因此体积比可能不适于作为PSNs的肿瘤特征，正如Takenaka等^[12]^研究体积对Ia期PSNs预后的影响时仍采用CTR作为观察变量。

### 3.2 病理亚型与三维平均CT值的关系

Son等^[13]^研究者发现，CT值可作为IAC的独立预测因素，在肺腺癌向浸润性病变的进程中，肺组织内局部肺间质增厚，癌细胞向肺泡内填充导致含气量减少乃至塌陷，肿瘤密度不断增加，影像学上表现为CT值增加。本研究中，实性成分和磨玻璃成分的3Dm-CT在两组间的显著差异也印证这一观点，两种成分的3Dm-CT均与病理亚型风险级别呈正相关性。同时，还有学者对各病理亚型的CT值做了鉴别工作^[14]^，发现CT值为-520 Hu可作为区分浸润前癌与浸润腺癌的截断值。而这一截断值虽与本研究PSNs中磨玻璃成分3Dm-CT的截断值为-509 Hu相近，但本研究的结论为当磨玻璃成分3Dm-CT > -509 Hu或实性成分3Dm-CT > -35 Hu时，PSNs的病理亚型为non-LPA可能性大。

Eguchi等^[15]^在测量肿瘤CT值方面，建议用感兴趣区域法（region of interest, ROI），即人为多次选取区域内的点获得每点的实际CT值，最后求平均CT值，该方法虽简单易行，但对于PSNs而言，区域内密度不均，人为选择随机性大，可能造成较大偏倚。而本研究针对PSNs，采用3Dm-CT概念作为客观量化指标，利用软件可极大降低ROI法造成的人为选择偏倚，同时也充分暴露了区域内磨玻璃和实性两种不同成分的细节。

### 3.3 病理亚型与SUVmax的关系

PET/CT作为高端医学影像检查方法，在目前医学领域中的应用越来越广泛，它既可以正确地表示出病变的解剖构造，还能检测^18^氟代脱氧葡萄糖（^18^F-flurodeoxyglucose, ^18^F-FDG）在肿瘤内的代谢水平以判断肿瘤良恶性，且在一定范围内代谢水平越高，恶性程度越大。Eriguchi等^[11]^分析了225例cIa期肺腺癌，其中包含了77例纯实性结节，发现截断值为SUVmax=1.9时可区分LPA与non-LPA，而本研究的截断值为SUVmax=1.26，截断值小于上述研究考虑可能因本研究样本量相对不足和纳入病例仅为PSNs所致。尽管如此，本研究也印证了SUVmax与肺腺癌病理亚型呈现一定关联性，在表现为PSNs的早期肺腺癌中，当SUVmax > 1.26时，病理亚型考虑为non-LPA可能性大。

### 3.4 两种影像学方式的联合预测价值

Fu等^[16]^进行了2, 010例I期肺腺癌大样本量研究，发现PSNs肺腺癌病理亚型与预后显著相关。据目前指南，建议所有浸润性非小细胞肺癌行肺叶切除术，但LPA的预后较好且与AIS、MIA相似，若在术中冰冻病理未能明确具体病理亚型，仅凭IAC就行肺叶切除，势必造成医疗资源浪费和患者器官功能的无谓损害。因此，术前对肿瘤病理亚型的预判十分必要，可直接影响术中决策。PET/CT联合CT三维重建，可评估PSNs的形态特征和代谢状态，同时也能综合各自区分良恶性的特点，优势互补，从而提高其准确性。本研究联合分析了两种方法的参数（肿瘤体积、磨玻璃成分体积、实性/磨玻璃3Dm-CT、SUVmax），发现其对早期PSNs病理亚型有良好预测价值（AUC=0.835）。

### 3.5 不足与展望

Mimics软件主要用于肺段切除术前的肺结节定位，其在对微小结节精确度的解析上虽略逊于诸多仪器设备自带的专业软件，但本研究所采用的解析方法与其他软件在本质上较为一致。在IT领域高度发达的今天，本研究重建参数获取的手动操作方法很容易实现全自动化，可极大节省解析时间。若本研究的结果在未来得到高级别证据印证，能应用于临床，在目前大量PSNs的诊疗过程中，术前即可对病灶病理亚型、预后有所判断，可提前制定更精准更个性化的围术期策略，减少手术相关风险，使患者最大获益。

本研究作为回顾性研究且因样本量限制，结果存在不同程度的偏倚。同时，本研究样本虽选取时间跨度大，但主要集中在2018年-2019年，生存信息获取年限不足，因此未行生存分析。后续将扩大样本量，充分获取生存信息，同时联合多中心进一步论证。
